# Efficacy of Trauma Catheter and Mushroom Tip Catheter in Evacuation of Chronic Subdural Hematoma and Complications of Drain Placement

**DOI:** 10.7759/cureus.5123

**Published:** 2019-07-11

**Authors:** Harjyot Toor, Ira Bowen, Bailey Zampella, Gohar Majeed, Christopher Elia, James A Berry, Shokry Lawandy, Rosalinda Menoni, Dan E Miulli

**Affiliations:** 1 Neurosurgery, Riverside University Health System Medical Center, Moreno Valley, USA; 2 Neurosurgery, Arrowhead Regional Medical Center, Colton, USA

**Keywords:** chronic subdural hematoma, evacuation, catheter, twist drill craniostomy, closed drainage system

## Abstract

Objective

The aim of this study was to assess the efficacy and complications of trauma catheter versus mushroom tip catheter placement in the evacuation of chronic subdural hematoma via twist drill craniostomy with closed system drainage.

Background

Chronic subdural hematoma (cSDH) is one of the most frequent neurosurgical pathologies in patients >70 years of age with an estimated incidence of 8.2 per 100,000 people per year. The most common risk factors for cSDH are advanced age, alcohol abuse, seizures, cerebrospinal fluid (CSF) shunts, coagulopathies, blood thinners, and patients at risk for falling. Twist drill craniostomy can be performed at the bedside under local anesthesia, making it an attractive treatment option, especially in poly-morbid patients who are poor surgical candidates. A closed drainage system is placed at the time of surgery to allow continuous drainage and promote postoperative brain expansion. Despite the increasing prevalence, limited literature exists to guide surgical management, particularly in terms of drain management and selection of catheter.

Methods

This is a retrospective review of 205 patients from January 2007 to May 2017 at two-level high volume centers for the evaluation and treatment of cSDH. Inclusion criteria include patients >18 years of age with the radiographic presence of a subdural hematoma for greater than three weeks. All patients were managed with either a trauma catheter or mushroom tip catheter. All patients received computed tomography (CT) of the head without contrast prior to subdural drain placement and within 24 hours after subdural drain removal. Exclusion criteria include patients <18 years of age and patients with depressed skull fractures, vascular malformations, subdural empyema, subdural hygroma, or who initially underwent open craniotomy or burr-hole craniotomy.

Results

Drain efficiency in evacuating the cSDH was assessed using both radiographic and clinical markers. Analysis of 205 patients treated by twist drill craniostomy and the subsequent closed system drainage utilizing either the mushroom tip catheter or trauma catheter revealed that neither catheter was superior in producing a statistically significant change in the maximum thickness of the cSDH (*p *= 0.35) and midline shift (*p *= 0.45). Furthermore, when assessing patients clinically via utilization of the Glasgow Coma Scale (GCS), both the trauma catheter and the mushroom catheter did not show a statistically significant difference in improving GCS after the evacuation of the cSDH (*p *= 0.35). Neither catheter was associated with an increased incidence of hemorrhage with drain placement requiring open surgery (*p* = 0.12), need for additional drain placement (*p *= 0.13) or decline in GCS with intervention (*p* = 0.065).

Conclusion

Analysis of the 205 patients treated by twist drill craniostomy with closed system drainage for the evacuation of chronic subdural hematoma utilizing either the mushroom tip or trauma catheters revealed that neither catheter was statistically significant in radiographic or clinical improvement in evacuating cSDH. Furthermore, neither catheter was found to be associated with an increased complication risk.

## Introduction

Chronic subdural hematoma (cSDH) is one of the most frequent neurosurgical pathologies in patients >70 years of age with an estimated incidence of 8.2 per 100,000 people per year [1-2]. It is typically caused by shearing of bridging veins that course along the cortical surface of the brain forming an extra-axial fluid collection that fails to be absorbed and is often associated with trauma. Elderly patients are particularly susceptible to developing cSDH, as progressive brain atrophy leads to an increased size within their subdural space and tension along the bridging vessels. Other major associated risk factors include alcohol abuse, seizures, cerebrospinal fluid (CSF) shunts, coagulopathies, antiplatelet/anticoagulant use, and patients at risk for falling. With an aging population and increasing use of antiplatelet and anticoagulation therapies, a rise in cSDH prevalence is anticipated [3].

Patients with cSDH can be managed both surgically and non-surgically as the natural progression of the disease process allows the blood products to breakdown and be reabsorbed over time. However, it is not uncommon for patients to become symptomatic secondary to mass effect and midline shift provoked by the cSDH or brain irritation from subdural hematoma (SDH) breakdown products. Symptoms can vary from headache (35%), weakness (7.5%), lethargy (12%), confusion or disorientation (9.2%), coma (4.2%), and increased frequency of falls [1,4]. Furthermore, some patients may continue to have persistent cSDH despite an extended period of time as highly vascularized membranes encapsulate the subdural fluid collection and prevent its reabsorption. Surgical options for the treatment of cSDH that have failed non-surgical management include craniotomy, burr hole craniotomy, or twist drill craniostomy with continuous closed system catheter drainage [2,5-9].

Twist drill craniostomy, developed at the Montreal Neurological Institute by Dr. William Cone and associates, was first reported as a diagnostic procedure in 1966 [1]. In 1977, Tabaddor and Shulman proposed that slow continuous drainage following twist drill craniostomy and placement of catheter offered substantial advantages in the treatment of cSDH compared to the previously reported methods including craniotomy with membranectomy or burr hole. Camel et al. observed an 86% clinical improvement in 102 patients after bedside twist drill craniostomy with a continuous closed system catheter drainage [1]. Furthermore, patients were noted to have decreased morbidity and length of hospitalization [1,10-12]. However, despite increasing cSDH prevalence, limited evidence exists to guide surgical management, particularly in terms of drain management and catheter selection [10-13]. The purpose of this retrospective, two-center study is to examine patients undergoing twist drill craniostomy with a continuous closed system catheter drainage for cSDH, comparing efficiency, complications, and outcome in patients receiving either trauma catheter versus mushroom tip catheter.

## Materials and methods

**Figure 1:**
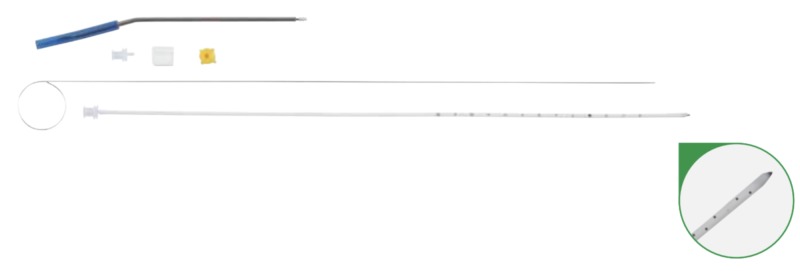
Integra TraumaCath Ventricular Catheter Set

**Figure 2:**
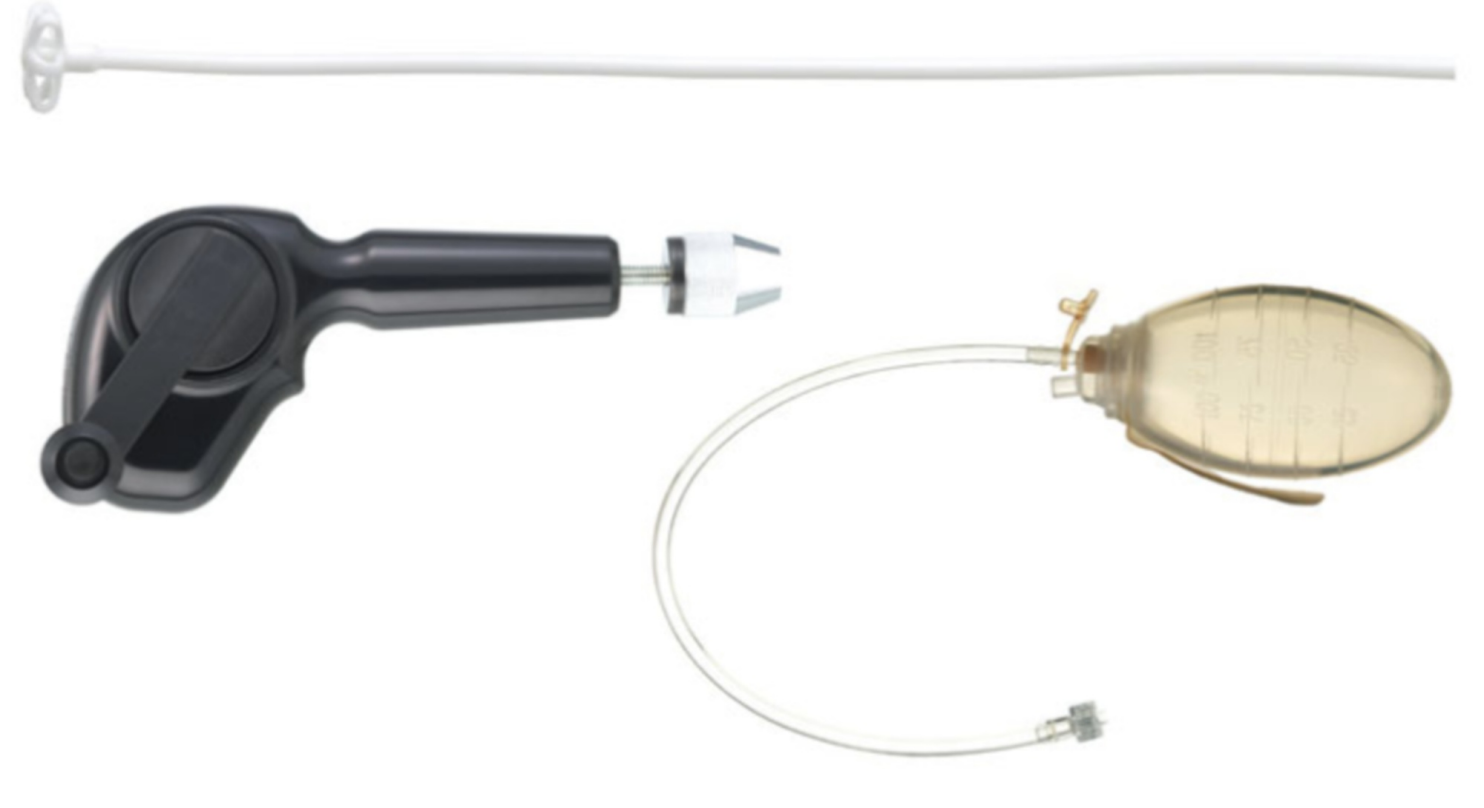
Integra NeuroSciences Subdural Evacuation Catheter Convenience Kit including the mushroom tip catheter

From January 2007 to May 2017, 205 patients were admitted to the Neurosurgery service, or the Neurosurgery service was consulted for the evaluation and treatment of cSDH at two high-volume trauma centers. This study was approved by the Institutional Review Board (IRB) at both centers. All patients included in the study were over the age of 18 years, possessed a subdural hematoma with a component of chronicity (minimum of three weeks) as defined radiographically as hypodense, isodense, or mixed density subdural fluid collection and were initially managed surgically with the placement of either a trauma catheter (Integra NeuroSciences TraumaCath^TM^ Ventricular Catheter Set, REF Catalog Number INS8420 [Integra Lifesciences Corporation, Plainsboro Township, NJ, USA]) or a mushroom tip catheter (Integra NeuroSciences Subdural Drainage Catheter Kit, REF Catalog Number 951310 [Integra Lifesciences Corporation]) via a bedside twist drill craniostomy (Figures 1, 2) [3].

Patients with depressed skull fractures, vascular malformations, subdural empyema, subdural hygroma, or who initially underwent open craniotomy or burr-hole craniotomy for evacuation of cSDH were excluded from this study. All twist drill craniostomies were performed at the bedside in either the neurological intensive care unit or the emergency department. A standard pre-procedural checklist was utilized, including administration of antibiotics within one hour of incision, loading dose of anti-epileptic medication, and correction of any platelet dysfunction or coagulopathy as necessary. The patient was placed in a supine position and the scalp was prepped in a sterile fashion. A 1-cm skin incision was made overlying the maximum thickness of the cSDH. A twist drill was used to create an orthogonal hole in the desired trajectory. The dura was opened using an eleven blade and then a subdural catheter, either a trauma or mushroom tip catheter, was inserted into the subdural space. The catheter was then connected to a Jackson Pratt suction bulb, permitting the contents of the subdural cavity to drain. Placement of the catheter was verified by computed tomography (CT) scan. 

Patients remained flat in bed with head turned side to side every hour to facilitate drainage, encouraged to perform incentive spirometry in an attempt to expand the brain, and serum sodium levels were kept at 130-135 mEq/L. All patients received a CT Head without contrast prior to subdural placement, immediately after subdural drain placement, at 48 hours after subdural drain placement, and after subdural drain removal. Furthermore, all patients were assessed neurologically with a Glasgow Coma Score (GCS) at the time of placement, and then at the time of hospital discharge.

The primary outcomes were a net change in maximum thickness in cSDH and midline shift (MLS) as noted on CT imaging and GCS. Secondary outcomes were failures in treatment and defined as the need for surgery for evacuation of hemorrhage associated with drain placement, need for additional drain, the decline in GCS, or new-onset seizures. Statistical analysis was performed with ANOVA and *t*-test analysis.

## Results

A total of 205 patients were included in this study, including 62 patients from Riverside University Health System (RUHS) and 143 patients from Arrowhead Regional Medical Center (ARMC). A total of 145 patients were managed with a mushroom tip catheter (44 at RUHS, 101 at ARMC) and 60 patients were managed with a trauma catheter (20 at RUHS, 40 at ARMC). In the mushroom tip catheter cohort, patients were of age 31 to 93 years with the average age being 68 years. Thirty-nine patients (26.9%) were female and 106 (73.1%) were male. In the trauma catheter cohort, patients’ age ranged from 43 to 91 years with the average being 65 years. Twelve patients (20%) were female and 48 (80%) were male. Of the patients in the trauma catheter group, 28 patients (46.7%) were Hispanic, eight (46.7%) were Caucasians, four (6.7%) were African Americans, and four (6.7%) were Asians. In the mushroom tip catheter group, 55 patients (37.9%) were Hispanic, 55 (37.9%) were Caucasians, 16 (11.03%) were African Americans, and 19 (13.1%) were Asians. There was no statistical significance between the two groups (Table 1). 

**Table 1:** Patient demographics

	Mushroom Tip Catheter n = 145	Trauma Catheter n = 60
Average Age	68	65
Age Range	31-93	43-91
Female	39 (26.9%)	12 (20%)
Male	106 (73.1%)	48 (80%)
Hispanic	55 (37.93%)	28 (46.7%)
Caucasian	55 (37.93%)	24 40%)
African American	16 (11.03%)	4 6.7%)
Asian	19 (13.1%)	4 (6.7%)

Two radiologic measures, maximum thickness of cSDH and midline shift (MLS), were employed to assess the efficiency of the catheter that was selected based on surgeon preference. The axial and coronal sequences of the CT head without contrast were used to identify the maximum thickness, diameter, of the extra-axial fluid collection including sulci effacement. Furthermore, the axial sequence of the CT head without contrast was used to identify the midline shift, if any was present. Midline shift was defined as deviation from the midline at the level of the foramen of Monro. All images were analyzed by a single observer to limit inter-observer variation. 

**Figure 3:**
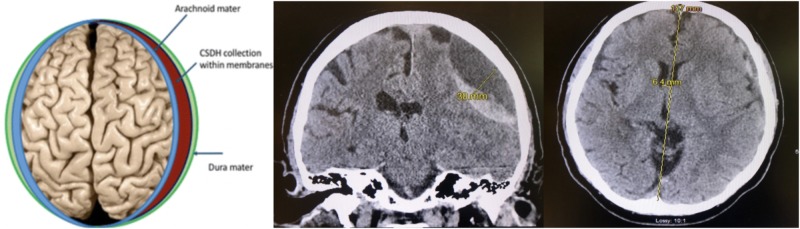
CT head illustrating maximum thickness and midlines shift of cSDH CT, computed tomography; cSDH, chronic subdural hematoma

For the mushroom tip catheter cohort, the average maximum thickness was 17.91 mm prior to subdural drain placement (range: 6.1 to 94 mm). At the time of drain removal, the average maximum thickness was 8.92 mm (range: 2 to 39 mm), leading to a net improvement of 8.98 mm in maximum thickness of the SDH. In the trauma catheter cohort, the average maximum thickness of the subdural hematoma prior to subdural drain placement was 13.87 mm (range: 5 to 24.8 mm). At the time of drain removal, the average maximum thickness was 9.28 mm (range: 1 to 32 mm), leading to a net improvement of 4.58 mm in maximum thickness of the SDH (Table 2).

**Table 2:** Radiographic characteristics of chronic subdural hematoma before and after drain placement CT, computed tomography

	Mushroom Tip Catheter n = 145	Trauma Catheter n = 60	Significance P-value
CT- Average maximum thickness prior to subdural drain placement	17.91 mm	13.87 mm	0.057
CT- Average maximum thickness at the time of subdural drain removal	8.92 mm	9.28 mm	0.070
Average net improvement in maximum thickness	8.98 mm	4.58 mm	0.35
CT- Average midline shift prior to subdural drain placement	6.74 mm	7.04 mm	0.125
CT- Average midline shift at the time of subdural drain removal	3.57 mm	4.79 mm	0.183
Average net improvement in midline shift	4.83 mm	4.97 mm	0.45

The second radiographic marker that was employed to assess for catheter efficiency was midline shift. Midline shift was measured at the axial slice identifying the foramen of Monro. In the mushroom tip catheter cohort, the average midline shift prior to subdural drain placement was 6.74 mm (range: 0 to 16 mm). At the time of subdural drain removal, average MLS was 3.57 mm (range: 0 to 26 mm), leading to an improvement of MLS by 4.83 mm. In the trauma catheter cohort, the average midline shift prior to the drain placement was 7.04 mm (range: 1 to 17.4 mm). At the time of subdural drain removal, average MLS was noted to be 4.79 mm (range: -2.2 to 13 mm), leading to an improvement in MLS by 4.97 mm.

The outcomes of using trauma versus mushroom tip catheter were also assessed clinically via the GCS prior to subdural drain placement and at time of hospital discharge. The neurological assessment was performed by neurosurgery resident and attending physicians. In the mushroom tip catheter cohort, the average GCS prior to subdural drain placement was 14.006. At the time of hospital discharge, the average GCS was 14.055 with a net improvement of 0.91 points in GCS. In the trauma catheter cohort, the average GCS prior to subdural drain placement was 13.56. At the time of hospital discharge, the average GCS was 13.64 with a net improvement of 0.70 (Table 3).

**Table 3:** Patient Glasgow Coma Scale before subdural drain placement and at the time of hospital discharge GCS, Glasgow Coma Scale

	Mushroom Tip Catheter n = 145	Trauma Catheter n = 60	Significance P-value
Average GCS prior to subdural drain placement	14.006	13.56	
Range of GCS prior to subdural drain placement	7-15	6-15	
Average GCS at the time of hospital discharge	14.055	13.64	
Range of GCS at the time of subdural drain removal	3-15	3-15	
Net improvement in GCS (points)	0.91	0.70	0.26

Four outcomes were assessed and classified as the failure of treatment, need for surgery, need for additional drain, the decline in GCS and new seizure onset after placement of the catheter. The need for surgery is defined as a conversion from bedside twist drill craniostomy with a closed drainage system to either open craniotomy versus burr hole craniotomy due to the need for evacuation of hemorrhage >1 cm maximum thickness or >5-mm midline shift associated with drain placement. The need for an additional drain was defined as any case in which a second catheter was needed to evacuate any or all residual cSDH as deemed appropriate by the supervising neurosurgeon. The decline in GCS from the time of drain placement to hospital discharge was considered a failure of treatment. Lastly, if a patient who did not present with seizures nor did they have a seizure history experienced new-onset seizures after insertion of the catheter (Table 4).

**Table 4:** Patient outcomes/failure of trauma versus mushroom tip catheter placement GCS, Glasgow Coma Scale *No patient included in this study had a subdural drain pulled out or fall out. All drain removals were performed at the discretion of the neurosurgery team. No infections occurred in these patients.

	Mushroom Tip catheter n = 145	Trauma Catheter n = 60	Significance p value
Failure of treatment- Need for surgery	40 patients (27.97%) - 12 patients refusing surgery (8.39%)	12 patients (19.35%) - 2 patients refusing surgery (3.22%)	0.12
Failure of treatment- Need for additional drain	11 patients (7.69%)	2 patients (3.22%)	0.13
Failure of treatment- Decline in GCS	10 patients (6.99%)	8 patients (12.90%)	0.065
New seizure onset after insertion of catheter	8 patients (5.51%)	6 patients (10%)	0.15

## Discussion

Chronic subdural hematoma (cSDH) is one of the most frequent neurosurgical pathologies, plaguing patients with varying symptoms, including but not limited to lethargy, somnolence, altered mental status, and increasing frequency of falls. Symptomatic patients or those that have failed non- surgical management can be managed surgically with either craniotomy with membranectomy, burr hole craniotomy, or twist drill craniostomy with a continuous closed system catheter drainage. Despite increasing cSDH prevalence, limited class one evidence exists to guide surgical management, particularly in terms of drain management and catheter selection.

Drain efficacy in evacuating the chronic subdural hematoma was assessed using both radiographic and clinical markers. Analysis of the 205 patients treated by twist drill craniostomy and the subsequent closed system drainage utilizing either the mushroom tip or trauma catheters revealed that neither catheter was superior in producing a statistically significant reduction in the maximum thickness of the cSDH (*p *= 0.35) and midline shift (*p *= 0.45). Furthermore, when assessing patients clinically via the GCS, both the trauma catheter and the mushroom tip catheter failed to be statistically significant in improving GCS with the evacuation of the cSDH (*p *= 0.35).

When assessing the various complications associated with twist drill craniostomy and closed system drainage, neither the mushroom tip catheter or the trauma catheter were found to produce a statistically significant increased incidence of hemorrhage with drain placement (*p *= 0.12), need for additional drain placement (p= 0.13) or decline in GCS with intervention (*p* = 0.065). Assessment of new seizure occurrence after drain placement revealed no change in percentage within either catheter group.

Consequently, when selecting between either the mushroom tip or the trauma catheter to be utilized in the twist drill craniostomy with a closed drainage system, neither catheter was found to be associated with increased drainage efficiency and efficacy as revealed by both radiographic and clinical markers. In addition, neither catheter was found to be associated with an increased complication risk. Thus one can infer that the efficacy of cSDH evacuation is rather dependent on the surgical decompression of the brain, rather than the catheter utilized to evacuate the extra-axial fluid collection.

Possible limitations to this study may include lack of recording of the exact duration of the subdural drain, lack of total volume of drainage at the time of placement of drain, lack of GCS at 24-48 hours post drain insertion. A longer-term GCS measurement may be necessary to assess the final outcome of the catheter intervention. Furthermore, this study did not investigate the presence of membranes in the cSDH and the role they may play in restricting catheter efficacy.

## Conclusions

Analysis of 205 patients treated by twist drill craniostomy and subsequent closed system drainage utilizing either the mushroom tip or trauma catheters revealed that neither catheter was superior in producing a statistically significant radiographic or clinical improvement in evacuating cSDH. Furthermore, neither catheter was associated with an increased complication risk.
